# Advanced MR techniques in glioblastoma imaging—upcoming challenges and how to face them

**DOI:** 10.1007/s00330-021-07978-8

**Published:** 2021-04-22

**Authors:** Timo A. Auer

**Affiliations:** 1 Department of Radiology, Campus Virchow-Klinikum, Charité - Universitätsmedizin Berlin, Augustenburger Platz 1, 13353 Berlin, Germany; 2 Berlin Institute of Health (BIH), Anna-Louisa-Karsch-Straße 2, 10178 Berlin, Germany

## Abstract

*• The management of gliomas has changed dramatically since the presentation of the revised WHO Classification of Tumors of the Central Nervous System in 2016 emphasizing the tumor heterogeneity based on their molecular profile.*

*• The need for a more noninvasive characterization of glioblastomas (GBM) by establishing reliable imaging biomarkers to predict patient outcome and improve therapy monitoring is bigger than ever.*

*• Multiparametric MRI, including promising newer techniques like electrical property tomography and mapping, may have the potential to provide enough information for intelligent imaging postprocessing algorithms to face the challenge by decoding GBM heterogeneity noninvasively.*

Assumptions about gliomas and their classification, including their most aggressive subtype, the glioblastoma (GBM), changed dramatically by the revised version of the WHO Classification of Tumors of the Central Nervous System in 2016 [1]. Additionally, as mentioned in the latest version of the European Association of Neuro-Oncology (EANO) guidelines, new insights were presented in the Consortium to Inform Molecular and Practical Approaches to CNS Tumour Taxonomy, further emphasizing the heterogeneity of GBMs based on their molecular profile [2]. Responding to these impulses, the EANO updated its consensus-based guideline for the management of adult patients with diffuse gliomas in 2020 [2]. As a result, the diagnostic disciplines have been confronted with the challenge to catch up with these new insights and provide novel diagnostic approaches.

Besides the clinical examination, magnetic resonance imaging (MRI) remains the gold standard for the diagnostic evaluation of affected patients and their response to treatment using the Response Assessment in Neuroradiology (RANO) criteria [3]. Nevertheless, even with the use of the highly standardized RANO criteria, it can be challenging to detect early progression. Additionally, therapy-associated effects like pseudoprogression can lead to over- or underestimation of the response [4]. It is to be expected that the new challenges posed by the modified classification and guidelines will further distort diagnostic assessment of GBM. Apart from the use of standardized MRI protocols, as reported elsewhere, there is a need for new, dedicated MR techniques that allow us to characterize the heterogeneity of GBMs and for intelligent image postprocessing solutions.

Basically, there are two scientific areas that hold promise for advancing the diagnostic characterization of GBM heterogeneity. One is the use and development of artificial intelligence (AI)–based image postprocessing algorithms including radiomics analysis, which may have the potential to significantly improve noninvasive diagnosis and subsequent management. The other are novel and advanced MRI techniques, which can be the ground truth for upcoming AI image postprocessing solutions and with doing so also multiplying their potential.

Furthermore, a multiparametric imaging approach may provide the desired prognostic tool that is both sufficiently accurate and easy to use in clinical practice. Therefore, several qualitative and quantitative MR techniques including diffusion-weighted imaging with apparent diffusion coefficient mapping (DWI/ADC), perfusion-weighted MRI, and volumetric approaches based on segmentation of different tumor subsections (mainly contrast-enhanced [CE], nonenhanced, and necrotic tissue) have been evaluated [5, 6]. An emerging MRI technique in all kinds of radiological fields including brain imaging are quantitative mapping sequences [7]. MRI mapping quantifies relaxation time at different echo times per pixel, thus providing information on tissue structure and composition. An interesting study by Blystad et al (2020) analyzed peritumoral edema of malignant gliomas using quantitative analysis of T1 and T2 mapping sequences, concluding that mapping techniques may be suitable for identifying peritumoral infiltrative growth. These results are in line with other interesting observations made by using mapping techniques in analyzing the peritumoral zones of high-grade gliomas [8].

Another promising candidate is electrical property tomography (EPT), which allows determination of tissue conductivity and can be quantified by means of a mapping-based analysis [9]. With EPT, it is possible to measure and visualize the regional conductivity of different brain and tumor regions as has already been tested for assessing the treatment response in a GBM in vivo study [9]. Although more studies are needed to confirm the effectiveness of the method and its validity in human studies, EPT may overcome the challenges of GBM imaging as it has the potential to yield more accurate information on the tumor microenvironment, also known as tumor “habitat,” especially in the setting of a multiparametric protocol.

An interesting review by Suh et al (2018) analyzed the value of multiparametric MRI for determining the treatment response in patients with GBM focusing on the discrimination of pseudoprogression from true progression. They concluded multiparametric MRI to be a promising tool for overcoming this pitfall, which is why the community is so excited about multiparametric approaches including EPT [10]. In this number of European Radiology, Park et al [11] report low conductivity on EPT demonstrating unique tumor habitats indicating tumor progression in GBM [11]. As one of the first in human studies, Park et al evaluated tumor habitats developed from EPT, DWI, and perfusion-weighted MRI in 60 patients with GBM who underwent concurrent chemoradiotherapy. The authors concluded that EPT reveals low-conductivity habitats that may improve the diagnosis of tumor progression in post-treatment GBM [11].

In conclusion, multiparametric MRI, including promising newer techniques like EPT and mapping, may have the potential to provide enough information for intelligent imaging postprocessing algorithms that may allow us to noninvasively decode GBM heterogeneity. If this can be accomplished, the next challenge will be to establish a protocol including techniques and software solutions smart and easy enough to implement in daily clinical routine.

## Declarations

### Guarantor

The scientific guarantor of this publication is Dr. Timo A. Auer (timo-alexander.auer@charite.de; Department of Radiology, Campus Virchow-Klinikum; Berlin Institute of Health (BIH); Charité - Universitätsmedizin Berlin)

### Statistics and biometry

No complex statistical methods were necessary for this manuscript.

### Methodology

• Editorial
